# IgA Vasculitis (Henoch-Schönlein Purpura) in a Seven-Year-Old Female Child With Recurrent Respiratory Infections

**DOI:** 10.7759/cureus.79360

**Published:** 2025-02-20

**Authors:** Mariam Sleem, Sheridan Padgett, Ahmed Rezk

**Affiliations:** 1 Medicine, Alabama College of Osteopathic Medicine, Dothan, USA; 2 Pediatrics, HCA Florida Gulf Coast Hospital, Panama City, USA

**Keywords:** glomerulonephritis (gn), henoch-schönlein purpura (iga vasculitis), non-specific abdominal pain, post-strep reactive arthritis, purpuric rash

## Abstract

A seven-year-old female child presented with a purpuric rash, joint pain, and a recent history of streptococcal pharyngitis. Initial symptoms included fever, throat pain, and vomiting, for which she received amoxicillin. Following treatment, the patient developed a rash and joint pain, prompting further evaluation. The physical examination revealed erythematous, edematous, and purpuric lesions on the extremities, along with periarticular swelling in the knees and ankles. Laboratory investigations showed hematuria, raising concerns for differential diagnoses, including IgA vasculitis, acute urticaria, acute glomerulonephritis, and idiopathic thrombocytopenic purpura. Treatment was adjusted with azithromycin and prednisolone. Despite these interventions, the patient's condition worsened, with new symptoms including fatigue, abdominal pain, and the spread of purpuric lesions. Although hospital admission was recommended, the patient's mother opted for care at an alternative facility. This case underscores the importance of early recognition and management of IgA vasculitis in pediatric patients and highlights potential links between prematurity and susceptibility to this condition.

## Introduction

IgA vasculitis is a rare disease, with an incidence of 3-27 per 100,000 individuals, predominantly affecting the pediatric population. It often presents after an upper respiratory tract infection, where the immune system mounts an exaggerated response, leading to systemic inflammation in the blood vessels. This inflammation can affect multiple organ systems, producing broad manifestations including palpable purpura, arthritis/arthralgia, abdominal pain, and kidney involvement in patients without underlying thrombocytopenia or coagulopathy [1]. More rarely, the vasculitis can affect the respiratory system. The most dangerous of respiratory manifestations is diffuse alveolar hemorrhage. Even without clinical manifestations, pulmonary changes may be noted on pulmonary function tests or radiology. Pathophysiology is thought to involve IgA deposition within the vessels of the alveolar septa, causing dysfunction in the alveolar-capillary membrane [2].

The case presented here involves a seven-year-old female patient with a history of recurrent respiratory infections, likely linked to her premature birth at 27 weeks gestation. Prematurity, particularly birth before 32 weeks, is associated with a higher risk of long-term respiratory morbidity, potentially contributing to recurrent infections in this patient. Compared to term delivery, birth before 32 weeks gestation carries a two-fold risk for long-term respiratory morbidity, whereas birth at moderate to late preterm (32.0-36.6 weeks) carries a 1.3-fold risk for long-term respiratory morbidity [3]. This case highlights the clinical manifestations, diagnostic considerations, and management strategies for IgA vasculitis while exploring the potential relationship between premature birth and susceptibility to this condition.

## Case presentation

A seven-year-old female child presented with complaints of a purpuric rash and painful joints. Her mother reported that two days prior, the child was treated for streptococcal pharyngitis with amoxicillin following symptoms of fever, throat pain, and vomiting. However, the patient developed a rash within 24 hours of starting the antibiotic. The mother presumed that they were insect bites, which progressively worsened in size and severity. The patient also experienced pruritus and reported difficulty walking due to joint pain.

The patient's medical history was notable for prematurity at 27 weeks gestation, recurrent respiratory infections (including pneumonia and streptococcal pharyngitis), and asthma. Her current medications included amoxicillin, Advair, albuterol, cetirizine, fluticasone, and montelukast. On physical examination, the patient appeared visibly distressed, tearful, and in pain. Her vital signs were as follows: BP 106/68 mmHg, HR 125 bpm, SpO_2_ 99%, and temperature 98.1°F.

**Figure 1:**
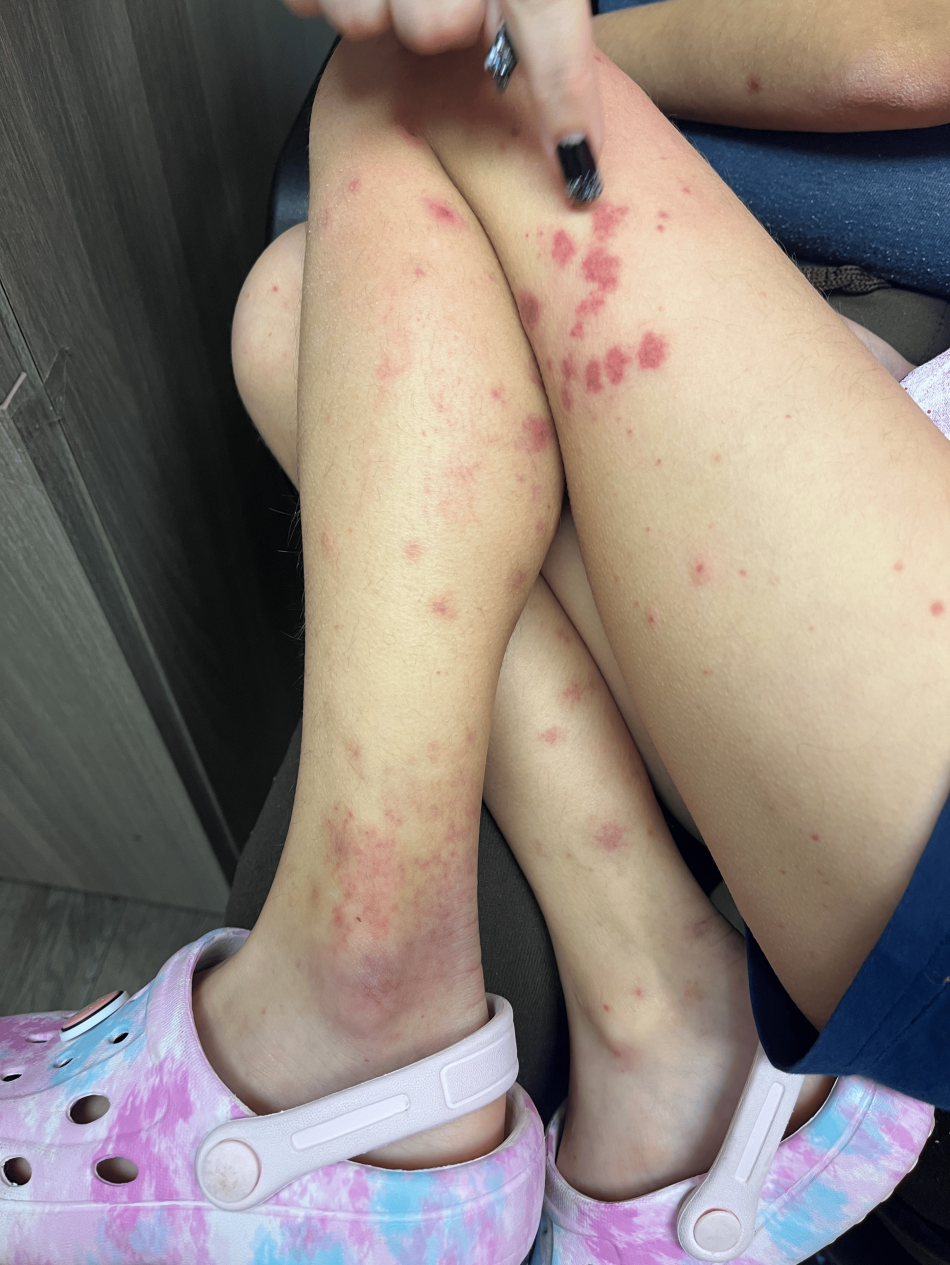
Lower extremities with purpura and swollen joints

**Figure 2:**
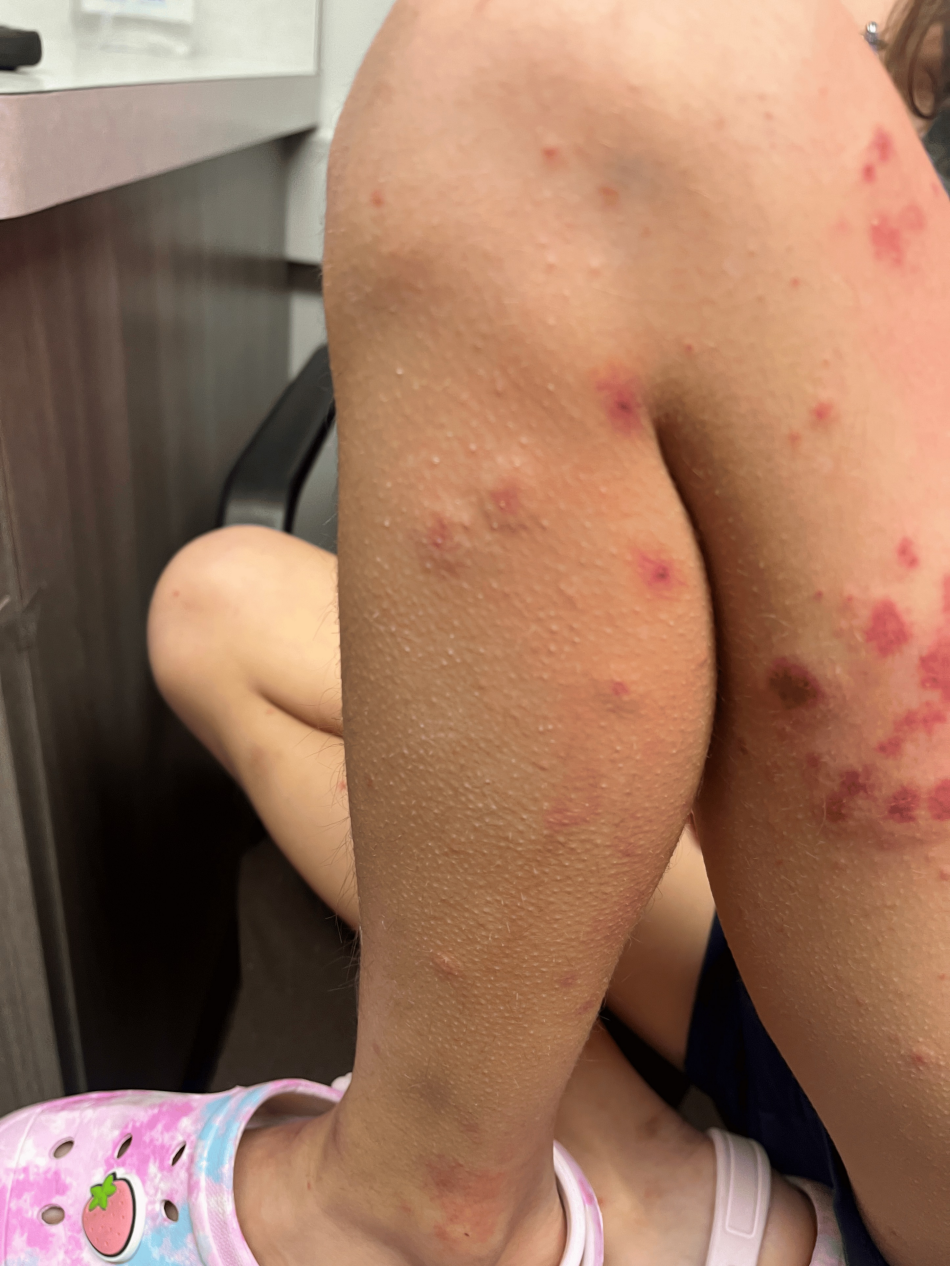
Left lower extremity with palpable purpura

**Figure 3:**
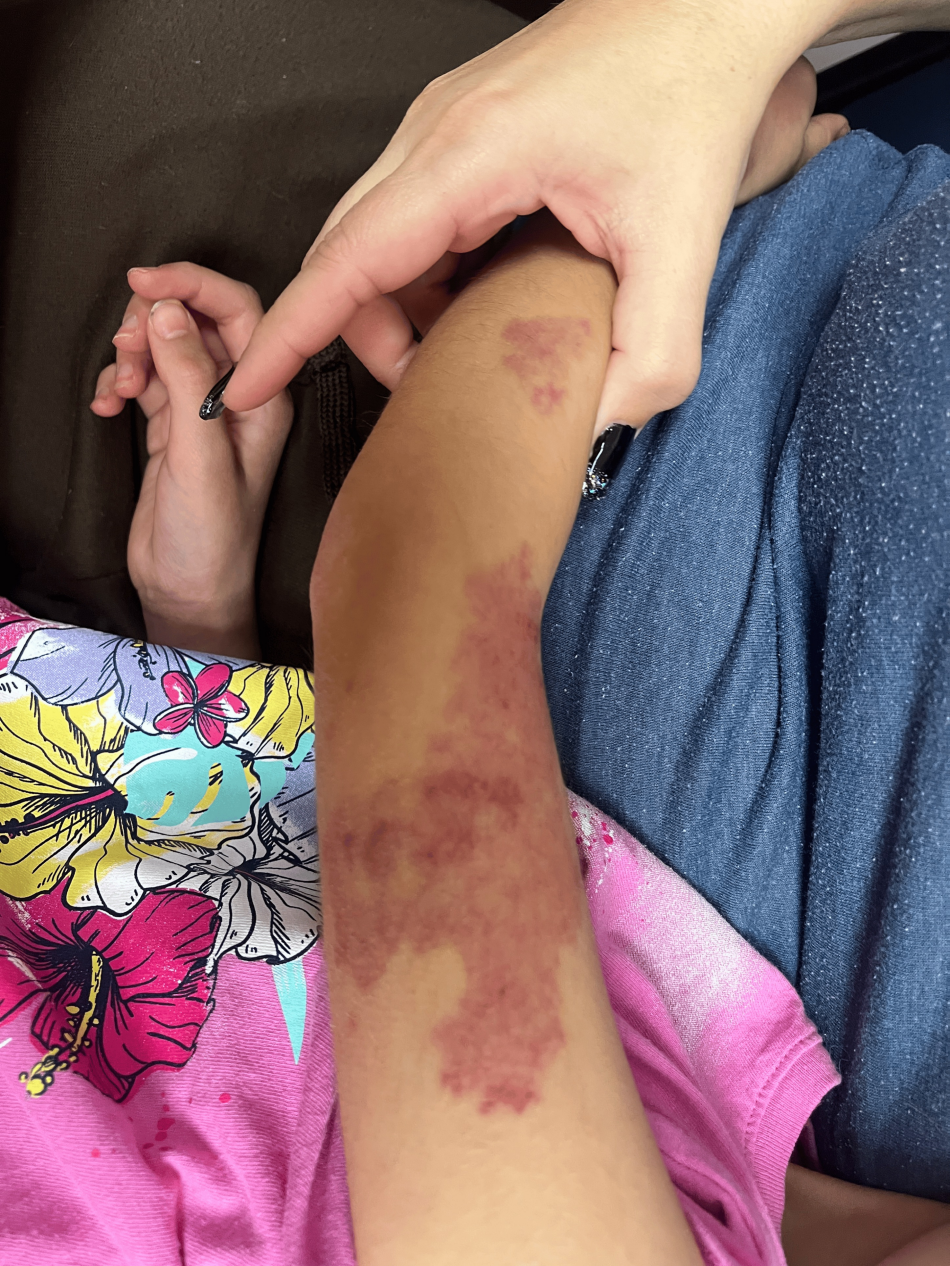
Palpable purpura in the left upper extremity

Examination revealed erythematous and edematous purpuric lesions on both upper and lower extremities (Figures 1-3), accompanied by periarticular swelling and tenderness in the knees and ankles. The rest of the physical examination was unremarkable, with clear lung fields, normal cardiac sounds, and no abdominal tenderness. Laboratory findings were significant for hematuria on urinalysis. Differential diagnoses included IgA vasculitis, acute urticaria, acute glomerulonephritis, and idiopathic thrombocytopenic purpura. Given the patient's recurrent respiratory infections, her antibiotic regimen was switched from amoxicillin to azithromycin, and corticosteroid therapy with prednisolone was initiated. A follow-up visit was scheduled for two days later, with instructions to monitor for hematuria or abdominal pain.

Upon returning for follow-up, the patient's mother reported worsening symptoms, including nausea, vomiting, fatigue, and new purpuric lesions behind the ears, on the face, and on the thighs. The child had also lost 5 lb since the previous visit. Despite these concerning developments, there were no changes in urine or stool color. The repeat physical examination confirmed the spread of the rash, although its character remained unchanged. Given the deterioration, hospital admission for close monitoring and further treatment was strongly recommended. However, the patient's mother declined hospitalization, opting instead for care at a more distant children's hospital.

**Figure 4:**
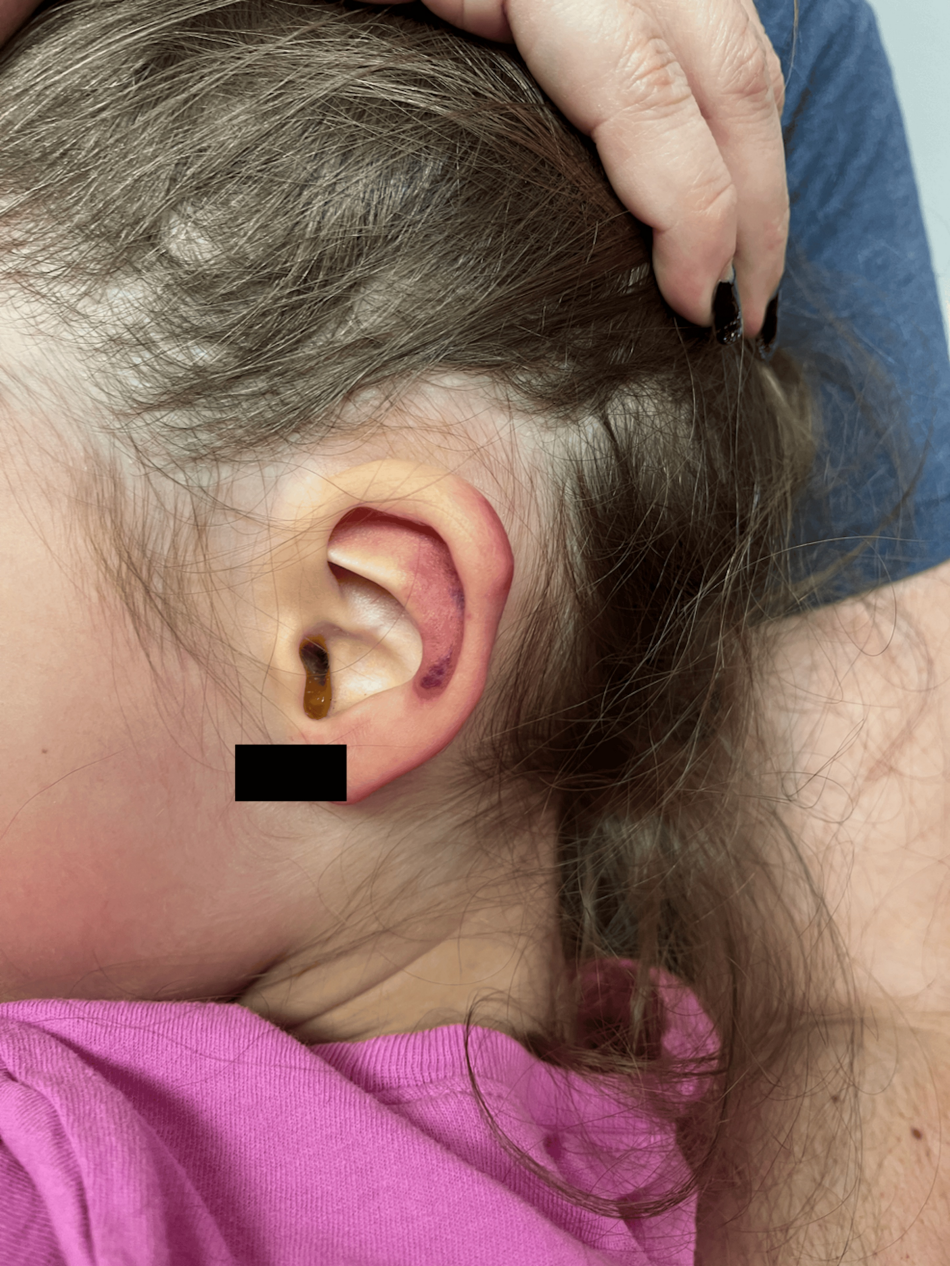
Purpura on the left ear

Upon returning to the office two days later, the patient's mother reported the child had experienced nausea, vomiting, appetite loss, fatigue, and abdominal pain. She also expressed concern about new lesions arising behind the ears (Figure 4).

## Discussion

IgA vasculitis, formerly known as Henoch-Schönlein purpura, frequently follows viral or bacterial infections, most commonly group A streptococcal infections. The underlying mechanism involves an exaggerated immune response that triggers systemic vasculitis, particularly affecting small vessels. Although the precise pathogenesis remains unclear, it is widely believed that immune complex deposition plays a central role in disease development.

The classic triad of symptoms includes palpable purpura, arthritis/arthralgia, and abdominal pain, with renal involvement also being a common complication. Early recognition is critical, as severe complications such as nephritis, intussusception, or gastrointestinal hemorrhage can arise without timely intervention.

This case raises several important considerations. The potential link between premature birth and heightened susceptibility to IgA vasculitis warrants further investigation. Prematurity is associated with long-term respiratory and immune system challenges, which may predispose individuals to exaggerated immune responses later in life [2]. Additionally, the potential respiratory manifestations raise alarm for the occurrence of the disease in those with prior existing pulmonary dysfunction. Recurrent streptococcal infections may play a role in increasing the risk of developing IgA vasculitis. Although IgA vasculitis is known to frequently follow streptococcal infections, the impact of recurrent infections on disease severity and recurrence is not well understood and deserves further exploration.

In this case of IgA vasculitis, the most pressing aspect of this presentation is to continue to monitor renal function and possible nephrology consultation. The patient presented to the clinic with hematuria, and therefore, a dose of oral steroids was prescribed. The patient did not return to the clinic and went to an out-of-network hospital due to the inability to ambulate and the further progression of the disease. It is likely that during her hospital stay, she was administered high-dose intravenous steroids, as they are often used and are successful in the treatment of glomerulonephritis with severe renal involvement [4]. The kidney is arguably the most serious organ involved in IgA vasculitis. In 2013, a double-blind, randomized trial was performed that compared corticosteroids with placebo. The study showed no benefit in reducing proteinuria 12 months after disease onset [4]. The pathophysiology behind kidney involvement is the deposition of IgA in the mesangium of the glomeruli, where blood filtration occurs, causing kidney manifestations such as hematuria [5]. This may lead to long-term kidney damage, and therefore, monitoring the kidney function in follow-up visits is crucial.

One limitation of this case is the lack of follow-up after the patient's transfer to another hospital, as mentioned above, limiting the ability to assess long-term outcomes and response to treatment. Nonetheless, the case emphasizes the need for prompt recognition and management of IgA vasculitis, particularly in pediatric patients with complex medical histories.

## Conclusions

IgA vasculitis is a rare disease that affects the pediatric population. The classic presentation includes palpable purpura, arthritis/arthralgia, abdominal pain, and kidney involvement. The case presented illustrates a classic case of IgA vasculitis in a patient whose past medical history may provide insight into the pathophysiology of the disease itself. This case highlights the importance of early diagnosis and management of IgA vasculitis, particularly in pediatric patients with underlying comorbidities such as prematurity and recurrent respiratory infections. Timely intervention can prevent serious complications, including renal failure, significant gastrointestinal bleeding, and extensive edema. This case offers potential areas for further exploration. Future research should explore the potential link between premature birth and the development of IgA vasculitis, as well as the role of recurrent infections in disease progression.
